# Accessibility of headache centers for patients suffering for cluster headache in Italy: too far from the patients’ needs

**DOI:** 10.1186/1129-2377-14-S1-P55

**Published:** 2013-02-21

**Authors:** P Rossi, C Geraci, C Tassorelli, G Nappi

**Affiliations:** 1 Headache Centre INI Grottaferrata, Italy; 2 AlCe Cluster Europe, Germany; 3 IRCCS C Mondino Pavia, Italy

## Background

Due to the extraordinary severity of pain, cluster headache (CH) warrants rapid diagnosis and appropriate treatment. The diagnosis of CH is simple, and rapid and effective treatments exist (injective sumatriptan and oxygen). In spite of this, clinical data have documented that CH is largely under-diagnosed and under-treated and it is common opinion that CH should be managed in a specialist setting. A fast access to headache services for CH patients is required to avoid delays to proper care. Aim of the study. To investigate the accessibility of the headache centers listed on the official websites of the two existing Italian societies involved into the study of headaches (SISC and ANIRCEF)

## Methods

Volunteers suffering for CH and serving as active members of Alleanza Cefalalgici Cluster (AlCe Cluster) contacted the Italian headache specialists searching for a fast access to a visit or for talking with the physicians. The primary outcome measure was the fast access to the headache specialist, defined as an access scheduled within 7 days from the contact. The secondary outcome measures were a) the possibility to talk with the physicians and b) a service measure of call-center efficiency (number of calls necessary to be answered). The study was conducted on April 2012.

## Results

151 headache centers were contacted in the study period. Fast access to a visit was allowed by 41 centres (31.7%, 33 covered by the national health system and 15 in private practice; in 16 cases a special referral of the GP certifying the urgency was requested). Only 9 centres (5.9%) gave to the patients the possibility to talk with the physician. 60 centres (39.7%)did not answered to the call (at least 3 call per day at different times for 5 five days).

## Discussion

The accessibility of headache centers for CH patients is inadequate and far from the patients’ needs for an irrational organization and a bad use of the technical and human resources. An inacceptable disparity emerges between different geographical areas.
